# Carapanolides J–L from the Seeds of *Carapa guianensis* (Andiroba) and Their Effects on LPS-Activated NO Production

**DOI:** 10.3390/molecules191117130

**Published:** 2014-10-24

**Authors:** Yuuki Matsui, Takashi Kikuchi, Takanobu Inoue, Osamu Muraoka, Takeshi Yamada, Reiko Tanaka

**Affiliations:** 1 Laboratory of Medicinal Chemistry, Osaka University of Pharmaceutical Sciences, 4-20-1 Nasahara, Takatsuki, Osaka 569-1094, Japan; 2 Faculty of Pharmaceutical Sciences, Kinki University, 3-4-1 Kowakae, Higashiosaka, Osaka 577-8502, Japan

**Keywords:** *Carapa guianensis*, andiroba, seeds, gedunin, phragmalin, limonoids, NO production

## Abstract

A novel gedunin and two novel phragmalin-type limonoids, named carapanolides J–L (compounds **1**–**3**) as well as a known gedunin-type limonoid **4** were isolated from the seeds of *Carapa guianensis* (andiroba). Their structures were determined on the basis of 1D and 2D NMR spectroscopy and HRFABMS. Compounds **1**–**4** were evaluated for their effects on the production of NO in LPS-activated mouse peritoneal macrophages.

## 1. Introduction

*Carapa guianensis* Aublet (Meliaceae)*,* known locally as andiroba, is widely distributed in the Amazonas State of Brazil and its wood is extensively used as commercial timber [1]. Andiroba is a tall rainforest tree that grows up to 40 m in height. The indigenous people in the Amazon have used andiroba in many ways for centuries, and virtually all parts of the tree, as well as the seed oil, are utilized. It can be found growing wild throughout the Amazon rainforest, typically on rich soils, in swamps, and also in the alluvial flats, marshes, and uplands of the Amazon Basin. This tree can also be found wild or under cultivation in Brazil in the Islands region, Tocantins, Rio Solimoes, and near the seaside. It is one of the large-leafed trees of the rainforest and can be identified by its large and distinctively textured leaves. The andiroba tree produces a brown, woody, four-cornered nut with a diameter of 3–4 inches that resembles a chestnut. Andiroba oil is a rich source of essential fatty acids including oleic, palmitic, stearic, and linoleic acids. It yields up to 65% unsaturated fatty acids and can contain approximatoly 9% linoleic acid. Andiroba oil extracts yield up to 65% unsaturated fatty acids and can contain approximately 9% linoleic acid. Extracts from its bark, flowers, and seeds have been used for centuries by the Amazonian people and exhibit various repellent [2], analgesic [3], anti-malarial [4], anti-inflammatory [5], anti-allergic [6], and antiplasmoidal [7] activities, as well as acute and subacute toxicities [8]. Our recent study on the components of the seed oil of *Carapa guianasis* revealed the structures of two new unusual 9,10-*seco*-mexicanolide-type limonoids, named carapanolides A and B [9], two novel carbon skeletal limonoids, named guianolides A and B [10], and carapanolides C–I [11]. We herein describe the isolation and structural determination of three novel limonoids **1**–**3**, named carapanolides J–L, and the effects of **1**–**3** and epoxyazadiradione (**4**) on the production of NO in LPS-activated mouse peritoneal macrophages. The structures of **1**–**3** were determined on the basis of NMR spectroscopy, including 1D and 2D (^1^H, ^1^H-COSY, NOESY, HSQC, HMBC) NMR, and FABMS. 

## 2. Results and Discussion

**Figure 1:**
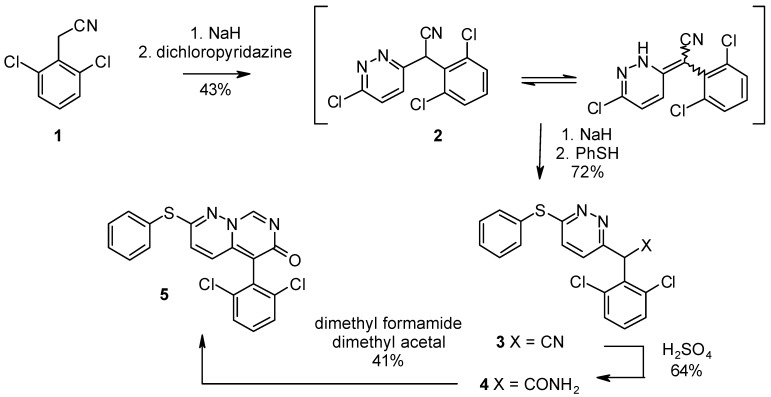
Chemical structures for compounds **1**–**4**.

The seed oil of *Carapa guianensis* (2.03 kg) was separated by silica gel column chromatography, medium-pressure liquid chromatography (MPLC), and reverse-phase HPLC to obtain three new limonoids **1**–**3** and a known limonoid **4**, which was identified as epoxyazadiradione (Figure 1) [12]. 

**Figure 2:**
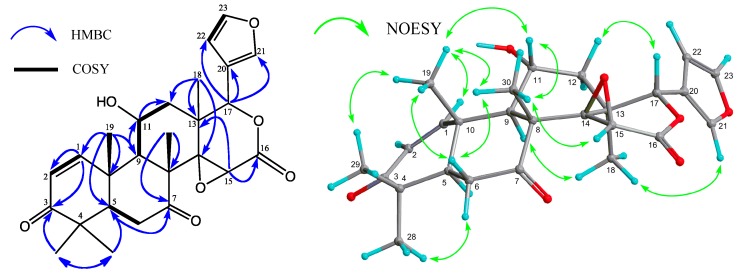
Key HMBC, COSY, and NOESY correlations for carapanolide J (**1**).

The molecular formula of carapanolide J (**1**) was determined as C_26_H_30_O_7_ ([M + H]^+^ *m/z* 455.2075) based on HRFABMS. The IR and UV spectra showed bands assignable to a hydroxy group (υ_max_ 3503 cm^−1^), a six-membered ring ketone (υ_max_ 1727 cm^−1^), and an α,β-unsaturated six-membered ring ketone [υ_max_ 1671 cm^−1^; λ_max_ 230 nm (log ε 3.85)]. The ^1^H and ^13^C NMR spectra (Table 1) exhibited signals assignable to five tertiary methyls [δ_H_ 1.16, 1.17, 1.21, 1.28, and 1.56]; two CH_2_ groups; five *sp*^3^ methine groups, including three oxymethine [δ_H_ 3.88 (s), 4.47 (ddd), and 5.49 (s)]; five *sp*^3 ^quaternary carbons, including an oxycarbon [δ_C_ 65.4 (s)]; an α,β-unsaturated six-membered ring ketone [δ_H_ 5.84, 8.24 (each 1H, d); δ_C_ 203.0 (s)]; a saturated ketone [δ_C_ 207.7 (s)]; δ-lactone [δ_H_ 5.49 (s); δ_C_ 166.4 (s)]; and furan ring [δ_H_ 6.39 (dd), 7.42 (t), 7.44 (m)]. In the HMBC spectrum, long-range correlations were observed between Me-18 (δ_H_ 1.21) and C-12, C-13, C-14 [δ_C_ 65.4 (s)], and C-17 [δ_C_ 77.6 (d)]; between Me-19 (δ_H_ 1.56) and C-1 (δ_C_ 160.2), C-5, C-9, and C-10; between Me-28 (δ_H_ 1.17) and C-3 (δ_C_ 203.0), C-4, C-5, and C-29; between Me-29 (δ_H_ 1.16) and C-3, C-4, C-5, and C-28; between Me-30 (δ_H_ 1.28) and C-7 (δ_C_ 207.7), C-8, C-9, and C-14; between H-11 (δ_H_ 4.47) and C-8, C-9, C-10, C-12, and C-13; between H-15 (δ_H_ 3.88) and C-8, C-13, C-14 and C-16 (δ_C_ 166.4); and between H-17 (δ_H_ 5.49) and C-12, C-13, C-14, C-16, C-18, C-20 [δ_C_ 120.0 (s)], C-21 [δ_C_ 141.1 (d)], and C-22 [δ_C_ 109.7 (s)] (Figure 2). An analysis of the ^1^H-^1^H COSY spectrum (H-1–H-2; H-5–H_2_-6; H-9–H-11–H_2_-12; and H-22–H-23) revealed the partial structure shown in Figure 2. The HMBC and ^1^H-^1^H COSY spectra revealed that **1** was a 11-hydroxy-7-deacetoxy-7-oxogedunin [13]. Selected NOESY correlations were shown in Figure 2. The secondary hydroxyl group at C-11 [δ_H_ 4.47 (ddd)] was determined to have an α (equatorial) orientation because significant NOEs were observed between H-11 and Me-19, and Me-30, while coupling constants were observed between H-11β and H-9α (*J*_11β,9α_ = 10.2 Hz); H-11β and H-12α (*J*_11β,12α_ = 13.5 Hz); H-11β and H-12β (*J*_11β,12β_ = 7.9 Hz). Therefore, compound **1** was determined to be 11α-hydroxy-7-deacetoxy-7-oxogedunin, which has been thus isolated in Nature for the first time.

**Table 1:** ^1^H (600 MHz) and ^13^C (150 MHz) NMR spectroscopic data of compound **1**.

Position		1				Position		1		
		^1^H *^a^* (*J*, Hz)		^13^C *^b^*				^1^H *^a^* (*J*, Hz)		^13^C *^b^*
1		8.24	d 10.3 (2)	160.2		14				65.4
2		5.84	d 10.3 (1)	124.9		15		3.88	s	54.1
3				203.0		16				166.4
4				45.6		17		5.49	s	77.6
5		2.21	dd 3.2 (6α), 14.6 (6β)	53.9		18		1.21	s	20.6
6	α	2.38	dd 3.2 (5), 13.8 (6β)	36.3		19		1.56	s	20.9
	β	2.93	dd 13.8 (6α), 14.6 (5)			20				120.0
7				207.7		21		7.44	m	141.1
8				53.4		22		6.39	dd 0.6 (21), 1.7(23)	109.7
9		2.45	d 10.2 (11)	51.3		23		7.42	t 1.7 (21, 22)	143.3
10				40.9		28		1.17	s	20.7
11	β	4.47	ddd 7.9 (12β), 10.2 (9), 13.5 (12α)	67.3		29		1.16	s	27.4
12	α	1.46	dd 13.5 (11), 13.8 (12β)	44.6		30		1.28	s	18.2
	β	2.21	dd 7.9 (11), 13.8 (12α)			11-OH		1.83	s	
13				38.0						

*^a^* Measured at 600 MHz in CDCl_3_; *^b^* Measured at 150 MHz in CDCl_3_. Assignments are based on HMBC spectrum.

**Figure 3:**
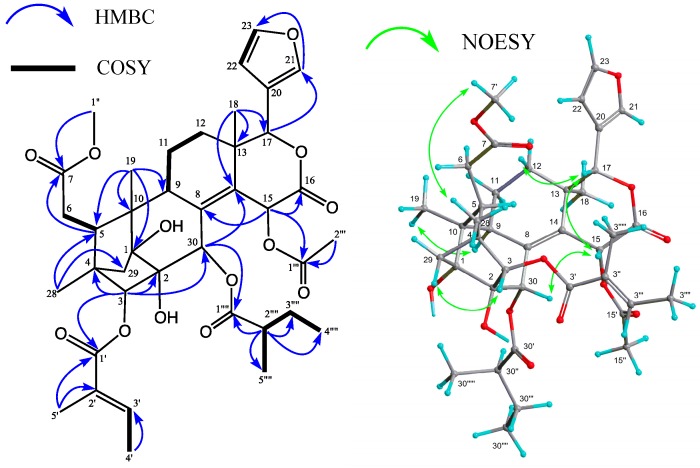
Selected ^1^H-^1^H COSY, HMBC and NOESYcorrelations for carapanolide K (**2**).

Carapanolide K (**2**), which was isolated as a colorless amorphous solid, had the molecular formula C_39_H_50_O_13_ ([M + Na]^+^; *m/z* 749.3152, calcd. for 749.3155) as determined by HRFABMS. The IR spectrum showed the presence of a hydroxyl at υ_max_ 3446 cm^−1^, and ester groups at υ_max_ 1766, 1735, and 1698 cm^−1^. The ^1^H -and ^13^C-NMR spectra (Table 2) indicated the presence of three methyls [δ_H_ 0.83, 1.09, 1.14 (each 3H, s)], an acetyl group [δ_H_ 2.07 (3H, s), δ_C_ 21.1 (q), 169.9 (s)], 2-methylbutanoyl group [δ_H_ 0.90 (3H, t), 1.13 (3H, d), 1.48 and 1.64 (each 1H, m), 2.38 (1H, m), δ_C_ 176.3 (s)], tigloyl group [δ_H_ 1.77 (3H, dd), 1.98 (3H, t), 7.14 (1H, qq); δ_C_ 168.5 (s)], methoxycarbonyl group [δ_H_ 3.72 (3H, s), δ_C_ 52.0 (q), 174.2 (s)], δ-lactone [δ_H_ 5.36 (1H, s), δ_C_ 80.3 (d), 167.8 (s)], two tertiary hydroxyl groups [δ_C_ 77.0 (s), 83.6 (s)], a tetrasubstituted double bond [δ_C_ 134.9 (s), 135.4 (s)], and furan ring [δ_H_ 6.47 (dd), 7.41 (t), 7.58 (t)]. In the HMBC spectrum, cross-peaks were observed between Me-18 [δ_H_ 1.09 (s)] and C-12, C-13, C-14 [δ_C_ 135.4 (s)], and C-17 [δ_C_ 80.3 (d)]; between Me-19 [δ_H_ 1.14 (s)] and C-1 [δ_C_ 83.6 (s)], C-5, C-9, and C-10; between Me-28 [δ_H_ 0.83 (s)] and C-3 [δ_C_ 88.2 (d)], C-4, C-5, and C-29; between H-3 [δ_H_ 4.73 (s)] and C-1, C-2 [δ_C_ 77.0 (d)], C-4, C-5, C-28, C-29, C-30 [δ_C_ 69.7 (d)], and C-3' [δ_C_ 168.5 (s)]; between H-5 [δ_H_ 2.88 (dd)] and C-1, C-3, C-4, C-6, C-7 [δ_C_ 174.2 (s)], C-10, C-19, C-28, and C-29; between H-30 [δ_H_ 5.41 (s)] and C-1, C-2, C-3, C-8 [δ_C_ 134.9 (s)], C-9, and C-30' [δ_C_ 176.3 (s)]. The positions of the hydroxyl, 2-methylbutanoyl, methoxycarbonyl, and tigloyl groups were identified by detailed ^1^H-^1^H COSY and HMBC correlations (Figure 3). In addition, the cross peaks between H-9 and H-30, and C-8 [δ_C_ 134.9 (s)]; between H-30, H-15, and C-14 [δ_C_ 135.4 (s)] revealed that compound **2** was a phragmalin-8(14)-ene derivative [14]. In the NOESY spectrum, significant NOEs (Figure 3) were observed between H-3 [δ_H_ 4.73 (s)] and H-29 *pro-S*, H-30, and Me-28; between H-5 [δ_H_ 2.88 (dd)] and Me-28 and H-30; between Me-18 and H-11α and H-12α; between Me-19 and H-11α, between H-15 and H-17β, H-30, H-3', H-5', and H-2'''; therefore, the 2-methylbutanoyl group at C-30 and acetoxy group at C-15 were all α while the tigloyl group at C-3 had a β orientation. The configuration of the 2-methylbutanoyl group at C-30 was deduced to be *R* because the chemical shift value and NOESY correlation were very similar to that of carapanolide F [11], which was determined as 2*R* by single-crystal X-ray diffraction analysis. 

**Figure 4:**
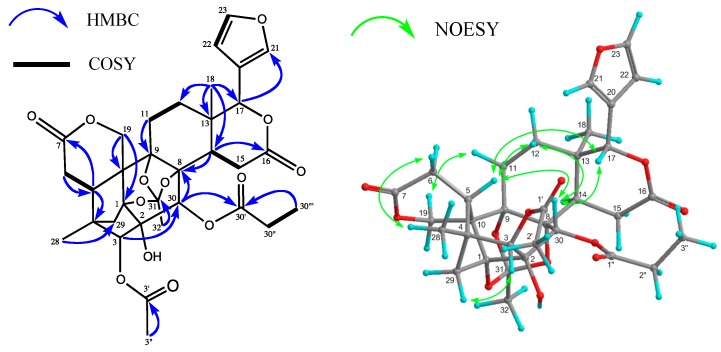
Key HMBC, ^1^H—^1^H COSY, and NOESY correlations of carapanolide L (**3**).

Carapanolide L (**3**) was obtained as a colorless amorphous solid, and its molecular formula was established as C_33_H_38_O_13_ ([M + H]^+^; *m/z* 643.2391, calcd. for 643.2391) by HRFABMS, implying 15 degrees of unsaturation. The IR spectrum showed the presence of a hydroxyl at υ_max_ 3352 cm^−1^, and ester groups at υ_max_ 1742 cm^−1^. The ^1^H- and ^13^C-NMR data indicated that eight of the 15 units of unsaturation came from two carbon–carbon double bonds and four ester carbonyls, including two lactone carbonyls. Therefore, the remaining degrees of unsaturation required **3** to be nonacyclic. The ^1^H- and ^13^C-NMR spectra of **3** (Table 2) indicated the presence of two tertiary methyls [δ_H_ 1.00, 1.13 (each s)], an acetyl [δ_H_ 2.19 (s); δ_C_ 21.6 (q), 170.4 (s)], propanoyl [δ_H_ 1.09 (3H, t), 2.36 (1H, dq), 2.39 (1H, dq); δ_C_ 8.6 (q), 27.8 (t), 172.8 (s)], and orthoacetyl group [δ_H_ 1.70 (s); δ_C_ 21.0 (q), 119.6 (s)], four methylenes, including an oxymethylene [δ_H_ 4.38 and 4.77 (each 1H, d), five *sp*^3^ methines, including three oxymethines [δ_H_ 4.66 (s), 5.35 (s), and 5.71 (s)], a furan ring [δ_H_ 6.41 (dd), 7.44 (t), and 7.48 (m)], seven *sp*^3^ quaternary carbons, including four oxycarbon [δ_C_ 79.5 (s), 85.4 (s), 86.3 (s), and 86.4 (s)], two ester carbonyls [δ_C_ 170.4, and 172.8 (each s)], and two lactone carbonyl [δ_C_ 169.8, and 171.1 (s)]. An analysis of the ^1^H-^1^H COSY spectrum of **3** revealed the partial structures shown in bold face in Figure 4. In the HMBC spectrum (Figure 4), cross-peaks were observed from Me-18 [δ_H_ 1.13 (s)] to C-12, C-13, C-14, and C-17 (δ_C_ 78.4); from Me-28 [δ_H_ 1.00 (s)] to C-3, C-4, C-5, and C-29; from H-30 [δ_H_ 5.71 (s)] to C-1 [δ_C_ 85.4 (s)], C-2 [δ_C_ 79.5 (s)], C-3 [δ_C_ 83.9 (d)], C-8 [δ_C_ 86.4 (s)], and C-9 [δ_C_ 86.3 (s)] from H-14 [δ_H_ 1.00 (s)] to C-8, C-9, C-12, C-13, C-15, and C-16 [δ_C_ 169.8 (s)]. Therefore, the planar structure of **3** was established as phragmalin-1,8,9-orthoacetate [13], and the positions of the hydroxyl, acetyl, and *n*-propyl groups were located at C-2, C-3, and C-30 by detailed ^1^H-^1^H COSY and HMBC correlations (Figure 3). In the NOESY spectrum, significant NOEs (Figure 3) were observed between H-3 [δ_H_ 4.73 (s)] and H-29 *pro-S*, H-30, and Me-28; between H-5β [δ_H_ 2.68 (dd)] and H-12β, Me-28, and H-30; between H-15β [δ_H_ 3.19 (dd)] and H-30; between H-17β [δ_H_ 5.35 (s)] and H-12β, H-15β, H-22, and H-30β, between Me-18 [δ_H_ 1.13 (s)] and H-11α, H-12α and Me-32. Therefore, the relative structure of **3** was established as shown in Figure 1.

**Table 2:** ^1^H-NMR and ^13^C-NMR data for compounds **2** and **3**.

Position		2			3		
		^1^H *^a^* (*J*, Hz)		^13^C *^b^*	^1^H *^a^* (*J*, Hz)		^13^C *^b^*
1				83.6			85.4
2				77.0			79.5
3		4.73	s	88.2	4.66	s	83.9
4				43.1			45.2
5		2.88	dd 1.2 (6B), 5.3 (6A)	37.5	2.68	dd 3.5 (6B), 5.5 (6A)	33.8
6	A	2.32	d 5.3 (5)	33.7	2.46	dd 5.5 (5), 17.6 (6B)	31.0
	B	2.33	d 1.2 (5)		2.66	dd 3.5 (5), 17.6 (6A)	
7				174.2			171.1
8				134.9			86.4
9		2.73	d 7.7	35.9			86.3
10				47.5			44.7
11	Α	1.70	m	18.3	1.85	dt 2.9 (11α), 14.7 (12α,β)	25.7
	Β	1.89	m		2.27	m	
12	α	1.05	m	28.5	1.48	m	29.4
	β	1.4	dt 3.2 (12α), 14.1 (11β)		1.38	m	
13				38.9			34.5
14				135.4	2.02	dd 2.0 (15β), 10.5 (15α)	42.8
15	α				2.70	dd 10.5 (14), 20.0 (15β)	26.4
	β	6.28	d 2.4	64.2	3.19	dd 2.0 (14), 20.0 (15α)	
16				167.8			169.8
17		5.36	s	80.3	5.35	s	78.4
18		1.09	s	16.7	1.13	s	20.0
19	α	1.14	3H, s	17.3	4.77	d 13.8 (19β)	68.8
	β				4.38	d 13.8 (19α)	
20				120.5			120.8
21		7.58	t 0.8 (22)	142.0	7.48	t 0.8 (22)	140.8
22		6.47	dd 0.8 (21), 1.6 (23)	109.9	6.41	dd 0.8 (21), 1.8 (23)	109.6
23		7.41	t 1.6 (22)	143	7.44	t 1.8 (22)	143.4
28		0.83	s	14.8	1.00	s	13.6
29	*pro-R*	1.58	d 11.0 (29* pro-S*)	39.8	1.80	d 11.1 (29* pro-S*)	38.3
	*pro-S*	1.86	d 11.0 (29* pro-R*)		2.25	d 11.1 (29 *pro-R*)	
30		5.41	s	69.7	5.71	s	70.0
31							119.6
32					1.70	s	21.0
1'				168.5			170.4
2'				130.0	2.19	s	21.6
3'		7.14	qq 7.0 (4'), 1.1 (5')	12.2			
4'		1.77	dd 1.1 (5'), 7.0 (3')	139.2			
5'		1.98	t 1.1 (3', 4')	14.5			
1''		3.72	s	52.0			172.8
2''	A				2.36	dq 7.5 (3''), 9.7 (2''B)	27.8
	B				2.39	dq 7.5 (3''), 9.7 (2''A)	
3''					1.09	3H, t 7.5 (2''A, 2"B)	8.6
1'''				169.9			
2'''		2.07	s	21.1			
1''''				176.3			
2''''	A	2.38	m	40.9			
	B						
3''''	A	1.48	m	26.5			
	B	1.64	m				
4''''		0.90	t 7.3 (3''''A, 3''''B)	16.4			
5''''		1.13	d 7.0 (2'''')	11.3			

*^a^* Measured at 600 MHz in CDCl_3_; *^b^* Measured at 150 MHz in CDCl_3_. Assignments are based on HMBC spectrum.

Physiological nitric oxide (NO) is involved in blood pressure regulation and blood flow distribution, whereas its overexpression may induce tissue injury, multiple organ dysfunction, and death, as well as systemic inflammatory responses in sepsis, such as hypotension, cardiodepression, and vascular hyporeactivity [15]. In the present study, four limonoids and l-NMMA, an inducible nitric oxide synthase (iNOS) inhibitor, were evaluated for their inhibitory effects on NO production in LPS-stimulated RAW264.7 cells (Table 3). To determine safe concentrations, the cytotoxicities of these limonoids against RAW 264.7 were assessed by the MTT assay. Compounds **1** and **3** showed non-toxicities at 3–100 μM, whereas **4** and **2** exhibited moderate cytotoxicities (IC_50_ **4**: 21.3 μM; **2**: 15.2 μM). In the inhibitory assay of NO production, compound **1** showed similar inhibitory activities (produced NO 83.4% at 10 μM; 61.8% at 30 μM; 16.8% at 100 μM) to the positive control, l-NMMA (produced NO 79.3% at 10 μM; 58.2% at 30 μM; 39.9% at 100 μM), with no cytotoxicities. Compound **4** exhibited superior inhibitory activities on NO production at non-toxic concentrations (produced NO 74.0% at 3 μM; 30.0% at 10 μM) to those of l-NMMA. These results suggested that compound **1** may be valuable as potential inhibitors of NO production.

**Table 3:** Inhobitory effects of NO production by limonoids from the seeds of *Carapa guianensis*.

Compound		Concentration (μM)				
		3	10	30	100	IC_50_ (μM)
**1**	Produced NO (%) ^a^	92.1 ± 1.5	83.4 ± 3.1	61.8 ± 1.8	16.8 ± 0.0	37.4
	Cell viability (%) ^a^	102.4 ± 0.8	101.0 ± 1.7	102.8 ± 0.6	103.4 ± 1.8	>100
**2**	Produced NO (%)	78.6 ± 1.9	58.3 ± 2.8	25.8 ± 7.0	7.1 ± 1.2	12.0
	Cell viability (%)	81.4 ± 0.8	65.6 ± 0.2	33.6 ± 6.3	0.4 ± 0.4	15.2
**3**	Produced NO (%)	95.6 ± 2.5	95.4 ± 1.2	95.4 ± 2.9	78.4 ± 2.3	>100
	Cell viability (%)	97.6 ± 0.6	97.3 ± 1.3	100.5 ± 0.4	94.4 ± 1.0	>100
**4**	Produced NO (%)	74.0 ± 5.0	30.0 ± 2.3	7.5 ± 1.0	3.9 ± 1.8	5.9
	Cell viability (%)	93.6 ± 1.4	99.7 ± 0.8	6.8 ± 0.3	3.3 ± 0.3	21.3
L-NMMA ^b^	Produced NO (%)	93.0 ± 3.3	79.3 ± 0.8	58.2 ± 2.4	39.9 ± 1.7	53.7
	Cell viability (%)	103.5 ± 0.5	102.0 ± 1.5	94.1 ± 1.4	96.5 ± 2.5	>100

^a^ Produced NO (%) and cell viability (%) were determined based on the absorbance at 570 nm, respectively, by comparison with values for DMSO (100%). Each value represents the mean ± standard error (S.E.) of three determinations. The concentration of DMSO in the sample solution was 2 μL/mL; ^b^ Positive control.

## 3. Experimental Section 

### 3.1. General Procedures

Melting points were determined on a Yanagimoto micro-melting point apparatus and were uncorrected. Optical rotations were measured using a JASCO DIP-1000 digital polarimeter. IR spectra were recorded using a Perkin-Elmer 1720X FTIR spectrophotometer. ^1^H- and ^13^C-NMR spectra were obtained on an Agilent vnmrs 600 spectrometer with standard pulse sequences, operating at 600 and 150 MHz, respectively. CDCl_3_ was used as the solvent and TMS, as the internal standard. FABMS were recorded on a JEOL-7000 mass spectrometer. Column chromatography was carried out over silica gel (70–230 mesh, Merck, Darmstadt, Germany) and MPLC was carried out with silica gel (230–400 mesh, Merck). HPLC was run on a JASCO PU-1586 instrument equipped with a differential refractometer (RI 1531). Fractions obtained from column chromatography were monitored by TLC (silica gel 60 F_254_, Merck). 

### 3.2. Plant Material

The oil of (2.03 kg) *Carapa guianensis* AUBLET (Meliaceae) was collected in the Amazon, Brazil, in March 2013. Kindly provided by Mr. Akira Yoshino (who is a representative of the NGO “Green Heart Love Amazon Project”). A voucher specimen (CGS-01-2) was deposited in the Herbarium of the Laboratory of Medicinal Chemistry, Osaka University of Pharmaceutical Sciences.

### 3.3. Isolation of Compounds **1**–**4**

The seed oil of *Carapa guianensis* AUBLET (Meliaceae) (2.03 kg) was dissolved in CHCl_3_ (1 L) and the CHCl_3_ solution was subjected to CC (silica gel 14 kg), to afford seven fractions: Fraction A (Fr. No. 1–85, 1.512 kg) was eluted with *n*-hexane-CHCl_3_ = 1:1, B (Fr. No. 86–179, 229.1 g) was eluted with CHCl_3_, C (Fr. No. 180–220, 29.3 g) was eluted with CHCl_3_-EtOAc = 5:1, D (Fr. No. 221–225, 13.2 g) was eluted with CHCl_3_-EtOAc = 2:1, E (Fr. No. 226–265, 84.5 g) was eluted with CHCl_3_-EtOAc = 2:1, F (Fr. No. 266–290, 25.3 g) was eluted with EtOAc, G (Fr. No. 291–315, 72.8 g) was eluted with EtOAc:MeOH = 5:1, and H (Fr. No. 316–333, 45.4 g) was eluted with MeOH. Residue D was rechromatographed over a silica gel column (CC) (230–400 mesh, 300 g) eluted with *n*-hexane- EtOAc (1:1) to give 13 fractions: D1 (Fr. No. 1–35, 1.52 g), D2 (Fr. No. 36–49, 0.81 g), D(3) (Fr. No. 50–88, 0.70 g), D(4) (Fr. No. 89–115, 0.53 g), D(5) (Fr. No. 116–130, 0.60 g), D(6) (Fr. No. 131–140, 0.52 g), D(7) (Fr. No. 141–205, 0.47 g), D(8) (Fr. No. 206–215, 0.51 g), D(9) (Fr. No. 216–220, 0.42 g), D(10) (Fr. No. 221–240, 0.40 g), D(11) (Fr. No. 241–250, 1.11 g), and D(12) (Fr. No. 251–313, 1.36 g). Fraction D(6) was subjected to CC (230–400 mesh, 40 g) eluted with *n*-hexane–EtOAc (3:1) to give an amorphous solid (24.1 mg) that was separated by HPLC (ODS, 75% MeOH, at 25 °C, flow rate 4.0 mL·min^−1^, UV = 220 nm, column 250 × 20 mm i.d., 5 μm) to give compounds **2** (6.2 mg) and **3** (1.79 mg). Fraction D(8) was subjected to CC (230–400 mesh, 40 g) eluted with *n*-hexane–EtOAc (3:1) to give an amorphous solid (34.0 mg) that was subjected to CC (230–400 mesh, 40 g) eluted with *n*-hexane–EtOAc (3:1) to give an amorphous solid that was purified by HPLC (ODS, 75% MeOH, at 25 °C, flow rate 4.0 mL·min^−1^, UV = 220 nm, column 250 × 20 mm i.d., 5 μm) to give compounds **1** (7.5 mg) and **4** (3.8 mg). Fraction D(9) was subjected to CC (230–400 mesh, 30 g) eluted with *n*-hexane–EtOAc (3:1) to give an amorphous solid (25.5 mg) that was separated by HPLC (ODS, 70% MeOH, at 25 °C, flow rate 4.0 mL·min^−1^, UV = 220 nm, column 250 × 20 mm i.d., 5 μm) to give compound **3** (6.2 mg).

### 3.4. Analytical Data

Compound **1**. Colorless crystals; mp 172–174 °C (from MeOH-CHCl_3_); ${\left[\alpha \right]}_{\text{D}}^{26}$ −18.7° (*c* 0.1, CHCl_3_); HRFABMS *m/z*: 455.2075 [M+H]^+^ (C_26_H_31_O_7_, calcd for 455.2080); UV (EtOH) λ_max_ nm (log ε): 230 (3.85), 237 (3.80), 248 (3.63); IR (KBr) υ_max_ cm^−1^; 3503 (OH), 2926, 1727 (O-C=O), 1671 (C=C-C=O); ^1^H- and ^13^C-NMR, see Table 1. FABMS* m/z* (rel. int.): 477 ([M+Na]^+^, 15), 455 ([M+H]^+^, 71), 83 (100).

Compound **2**. Colorless amorphous solids; ${\left[\alpha \right]}_{\text{D}}^{26}$ −72.2° (*c* 0.1, CHCl_3_); HRFABMS *m/z*: 749.3152 [M+Na]^+^ (C_39_H_50_O_13_Na, calcd for 749.3155); UV λ_max_ (EtOH) nm (log ε): 227 (4.19), 304 (3.98), 315 (4.00), 334 (3.72); IR (KBr) υ_max_ cm^−1^: 3446 (OH), 2967, 1766 and 1735, 1698; ^1^H- and ^13^C-NMR, see Table 2. FABMS* m/z* (rel. int.): 749 (33) ([M+Na]^+^, 3), 727 ([M+H]^+^, 100).

Compound **3**. Colorless amorphous solids; ${\left[\alpha \right]}_{\text{D}}^{26}$ −46.8° (*c* 0.1, CHCl_3_); HRFABMS *m/z*: 643.2391 [M+H]^+^ (C_33_H_38_O_13_, calcd for 643.2391); UV λ_max_ (EtOH) nm (log ε): 208 (1.26), IR (KBr) υ_max_ cm^−1^: 3352 (OH), 1742 (O-C=O); ^1^H- and ^13^C-NMR, see Table 2. FABMS* m/z* (rel. int.): 665 (33) ([M+Na]^+^, 12), 643 ([M+H]^+^, 100).

### 3.5. Determination of RAW264.7 Cell Proliferation

RAW264.7 cell proliferation was examined according to a method reported previously [16] with some modifications. Briefly, RAW264.7 cells (5 × 10^4^ cells in 100 μL) were seeded onto 96-well microplates, and incubated for 24 h. D-MEM (100 μL) containing test samples (final concentration of 100, 30, 10, or 3 μM) dissolved in DMSO (final concentration 0.2%) was added. After the cells had been treated for 24 h, the MTT solution was added. After 3 h of incubation, 20% sodium dodecyl sulfate (SDS) in 0.1 M HCl was added to dissolve the formazan produced by the cells. The absorbance of each well was read at 570 nm using a microplate reader. The optical density of vehicle control cells was assumed to be 100%.

### 3.6. Inhibitory Assay of NO Production

An inhibitory assay of nitric oxide production was performed according to a method reported previously [17] with slight modifications. Briefly, RAW264.7 cells (5 × 10^4^ cells in 100 μL) were seeded onto 96-well microplates, and incubated for 24h. D-MEM (100 μL) containing test samples (final concentration of 100, 30, 10, or 3 μM) dissolved in DMSO (final concentration 0.2%) and LPS (final concentration of 5 μg/mL) were added. After cells had been treated for 24 h, 50 μL of 0.1% *N*-(1-naphtyl)ethylenediamine in H_2_O and 50 μL of 1% sulfanylamide in 5% phosphoric acid were added. After being incubated for 30 min, the absorbance of each well was read at 570 nm using a microplate reader. The optical density of vehicle control cells was assumed to be 100%.

## 4. Conclusions

A novel gedunin and two novel phragmalin-type limonoids, named carapanolides J–L (compounds **1**–**3**), as well as a known gedunin-type limonoid **4** were isolated from the seeds of *Carapa guianensis* (andiroba). Their structures were determined by spectroscopic analyses. Compound **1** showed similar inhibitory activities (produced NO 83.4% at 10 μM; 61.8% at 30 μM; 16.8% at 100 μM) to positive control, l-NMMA (produced NO 79.3% at 10 μM; 58.2% at 30 μM; 39.9% at 100 μM), with no cytotoxicity. Known compound **4** exhibited superior inhibitory NO production activities at non-toxic concentrations (produced NO 74.0% at 3 μM; 30.0% at 10 μM) to those of l-NMMA. These results suggest that compound **1** may be a valuable potential inhibitor of NO production.
